# Chronic Spontaneous and Inducible Urticaria Associated With Mycoplasma pneumoniae Infection

**DOI:** 10.7759/cureus.18746

**Published:** 2021-10-13

**Authors:** George N Konstantinou, Ioannis Sagonas, Fani C Giannoula

**Affiliations:** 1 Department of Allergy and Clinical Immunology, 424 General Military Training Hospital, Thessaloniki, GRC

**Keywords:** antibodies, clarithromycin, complement fixation, rash, cold urticaria, dermographism

## Abstract

Acute childhood urticaria is a common disorder that has been associated with infections. In a few children, it may last for more than six weeks, thereafter it is characterized as chronic urticaria (CU). We report two cases, one suffering from chronic spontaneous urticaria and one chronic inducible urticarias (dermographism and cold urticaria). Both children had concomitant respiratory symptoms that were associated with *Mycoplasma pneumoniae* (MP) infection. Urticarias’ symptoms and signs were refractory to regular antihistamines dose but showed marked improvement or complete resolution following clarithromycin administration. CU response to antibiotics pointed strongly to a potential causative role of MP in the pathogenesis of chronic spontaneous and chronic inducible urticarias. It is not clear if MP was the etiopathogenic cause or just the trigger. Nevertheless, refractory to antihistamines urticarias associated with MP infection may respond to antibiotics, which should be considered as an alternative therapeutic approach.

## Introduction

*Mycoplasma pneumoniae* (MP) is a common pathogen that mostly affects the respiratory tract. Infection is often subclinical, but in patients, the clinical manifestations may vary from mild upper respiratory tract infection to bronchitis and pneumonia. MP infections are more common in young children and adolescents and tend to rise in summer and peak in winter [1].

Less commonly, MP can be responsible for extrapulmonary manifestations with or without respiratory symptoms, but only in a few a causal relationship has been established. These involve the central nervous system, the cardiovascular system, the blood, the joints, the gastrointestinal system, and the skin [2].

Dermatologic manifestations associated with MP infection include acute urticaria [2-5], erythematous maculopapular and vesicular exanthem [6], erythema multiforme [7], Stevens-Johnson Syndrome [8], MP-induced rash and mucositis [9], bullous papular purpuric gloves and socks syndrome [10], erythema nodosum and anaphylactoid purpura [3], urticaria multiforme [11], Henoch-Schonlein purpura and leukocytoclastic vasculitides [12, 13], and other rare cutaneous disorders [14].

We describe two cases with an urticarial rash associated with MP infection. The first case started with acute urticaria and shortly after experienced simultaneous onset of two inducible urticarias (cold urticaria and dermographism). The second case had chronic spontaneous urticaria.

## Case presentation

Case 1

A 13-year-old girl was referred to our Allergology Department for inspection of a four-day-old generalized urticarial rash (more than 50 urticarial lesions), partially responded to levocetirizine 5 mg qd. Simultaneously, she had a febrile (38.5°C) respiratory infection presenting abruptly with constitutional findings of malaise, myalgia, headache, sore throat and gradually worsening non-productive cough and wheezing. Chest X-ray radiography revealed diffuse bilateral, reticular, interstitial findings, and mild right pleural effusion.

Clarithromycin 500 mg qod for 14 days was empirically started, and levocetirizine 5 mg qd continued. Respiratory symptoms disappeared five days after. Acute urticaria lasted for four more weeks, but after clarithromycin initiation, the number of wheals and pruritus intensity were significantly improved within a few days.

Acute serum samples were negative for acute infection with i) *Clamidiophila pneumoniae*, ii) Influenza A and B, iii) Parainfluenza A and B, iv) Adenovirus, v) Epstein-Barr virus (EBV), vi) Cytomegalovirus (CMV), and v) Measles viruses. However, significant antibody response was found for MP with specific IgM: 1/2560 (Titers of ≥1:64 were interpreted as positive) (Complement Fixation Test, Virion/Serion, Würtzburg, Germany).

**Figure 1:**
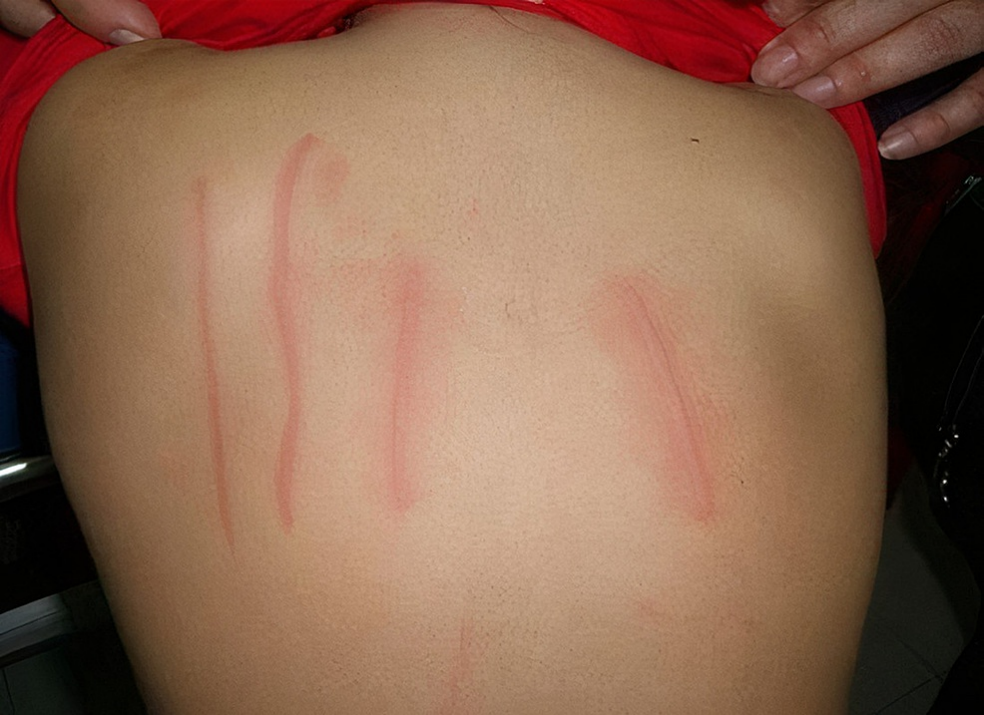
Titration of dermographism using a calibrated dermographometer

**Figure 2:**
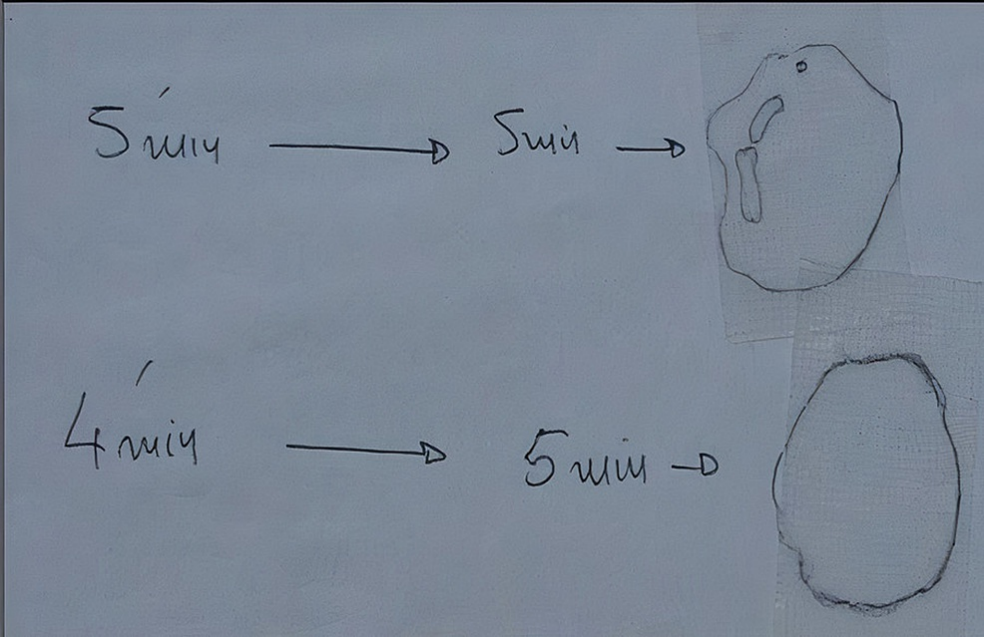
Cold stimulation tests at 4 and 5 minutes Reading was performed 5 minutes after removing the ice tube. External marked lines represent the flares reaction and the inner lines the wheals.

Two weeks after acute urticaria subsided, the girl complained about spontaneous linear whealing with pruritus and an urticarial rash related with swimming in the sea. At that time, she was re-evaluated and diagnosed as having symptomatic dermographism (wheal reaction with a calibrated dermographometer at approximately 30 gr/mm2) (Figure 1) and cold urticaria (positive Cold Stimulation Time Test at 5 minutes) (Figure 2). C3 and C4 were in the normal range, and cryoglobulins were not found. Both inducible urticarias were controlled with levocetirizine 5 mg qd for three weeks. Ten months later, she still experiences only symptomatic dermographism when off antihistamines; however, a minimum dose of 5 mg of levocetirizine once or twice a week was enough to control her symptoms.

Case 2

An 11 years old boy was referred to our Allergy Department for a seven-week-old generalized urticarial rash which appeared five weeks after a mild upper respiratory tract infection (URTI). A week before, his younger brother had bronchitis associated with MP that was successfully treated with clarithromycin. There were no fever, cough, or other associated constitutional symptoms present.

The patient was initially treated with levocetirizine dihydrochloride 5 mg qd, unsuccessfully. Two days after, an increase in dose to 5 mg bid resulted in pruritus control. However, urticaria persisted, and therefore, methylprednisolone 16 mg qd was introduced.

Physical examination revealed an alert, well-appearing boy in no apparent distress. His vital signs were unremarkable. On inspection, there were more than 10 urticarial lesions, ranging from 0.5 cm to 5 cm in diameter, most of which were present for more than 12 hours. The chest was clear to auscultation and the lymph nodes were normal in size. The abdomen was soft and non-tender, with no evidence of organomegaly. Chest X-ray radiography was normal. Complete blood count (CBC), basic metabolic panel, erythrocyte sedimentation rate (ESR) and C-reactive protein (CRP) came back normal.

In the context of his recent URTI infection and his brother’s bronchitis with MP, and the late onset of his urticaria and its persistence, he had been additionally tested for antinuclear antibodies (ANAs), antithyroglobulin antibodies (anti-TG abs), anti-thyroid peroxidase antibodies (anti-TPO abs), and cryoglobulins that yielded results within normal range. C3 complement level was 170 (normal range 65-150) and C4 level was only mildly high at 46.5 (normal range 12-45). Moreover, there was no evidence of EBV or CMV infection (undetectable virus-specific IgM and IgG antibody titers). However, the antibody response was found for MP with specific IgM 1/1024 (titers of ≥1:64 were interpreted as positive) (Complement Fixation Test, Virion/Serion, Würtzburg, Germany).

Clarithromycin 500 mg bid for 14 days was started, and levocetirizine was replaced with ebastine 20 mg bid for two weeks. The patient’s symptoms improved three days after the initiation of the new medication regimen, and therefore, methylprednisolone was tapered off gradually over a period of two weeks. The number and size of the wheals diminished as well as the duration of the urticarial rash. The patient experienced complete symptom resolution three weeks after the commencement of treatment. At a six-month follow-up visit, the patient was in good health, and the levels of C3, C4 and MP-specific IgM were within normal limits.

## Discussion

The marked improvement and complete resolution of symptoms seen in both patients following the empiric administration of an antibiotic agent could point strongly to a causative role of MP in the pathogenesis of acute and chronic spontaneous and inducible urticarias. In Case 2, the failure of standard treatment with an antihistamine and corticosteroids to control urticaria and the absence of any symptoms following a full course of antibiotic treatment, even after the discontinuation of methylprednisolone, further supports this hypothesis.

The exact role of MP in chronic urticaria is uncertain. To understand if any pathogenetic or clinical evidence could explain the link between MP and chronic urticaria (spontaneous or inducible), we conducted a systematic literature review. PubMed (for the MEDLINE database) and Scopus were searched by using a comprehensive search strategy ["(urticaria OR wheals OR hives OR dermographism) AND mycoplasma” Filters: Publication date to March 2021]. Inclusion criteria were: (1) clear distinction between chronic urticaria versus other allergies; (2) clear distinction of chronic spontaneous or idiopathic urticaria as opposed to acute urticaria; (3) studies in humans; and (4) published articles written in the English language. Additional potentially relevant studies were scrutinized in the reference lists of the eligible publications. Interestingly, only four relevant articles were identified.

Lim et al. reported that in 6.8% of adult patients, who presented at a private dermatology practice with chronic urticaria, there was evidence of acute MP infection. Nevertheless, the question of whether patients with acute urticaria and an untreated MP infection are at greater risk for developing chronic urticaria remains intriguing yet unanswered [15].

Gómez et al. found that nearly 40% of patients in a Latin American chronic urticaria registry tested positive for *Mycoplasma*-specific IgG (26). Unfortunately, the absence of a normal control population without urticaria renders the drawing of conclusions about the pathogenetic role of MP in chronic urticaria an elusive task [16].

Doeglas HM et al. reported that cold urticaria is serologically associated with complement-fixing antibody titers to MP and suggested no causal relationship but a basic immuno-regulatory defect of unknown origin [17].

Lastly, Yong SB et al. conducted a nationwide, population-based retrospective cohort study in Taiwan in which they identified an increased risk for acute urticaria among patients 20-59 years old with MP infection. This was not the case in patients <20 years of age, suggesting that patient-specific or idiosyncratic reasons may explain susceptibility [18]. In this study, the incidence of progression of acute urticaria to chronic was not examined.

Although the preferable method for MP infection diagnosis is polymerase chain reaction (PCR) from nasopharyngeal or oropharyngeal swab samples, serology (MP IgM and IgG ELISA or complement fixation test [CFT]) is a reasonable alternative with excellent specificity up to 97%, especially for the IgM determination with CFT [19].

According to the conducted systematic review, Case 1 is the first MP-associated dermographism in the literature. However, this developed cold urticaria, as well. More than one inducible urticarias can coexist in the same patient and may have a common pathophysiological background that can explain the simultaneous or sequential onset.

It has been suggested that most cases of childhood urticaria may be related to infections as associated triggers or even as etiopathogenic factors [20]. It can be hypothesized that infection with MP in genetically susceptible individuals may trigger immunological responses leading to autoimmune dysregulation that could explain the pathogenesis of chronic spontaneous or inducible urticarias.

## Conclusions

Chronic urticaria and prior upper or lower respiratory illnesses have been pathophysiologically linked. *Mycoplasma pneumoniae* infection may cause or trigger urticaria, especially in susceptible individuals. In the cases with recent respiratory infection and persistent urticaria, refractory to standard doses of antihistamines, serological examination of common respiratory pathogens is suggested. Appropriate antibacterial treatment should be considered in MP seropositive cases as it may constitute an alternative therapeutic option to control or even treat urticaria.
